# Effect of Dietary Supplementation with Salmon Oil on Canine Frozen–Thawed Semen

**DOI:** 10.3390/vetsci12090797

**Published:** 2025-08-23

**Authors:** Chiara Milani, Marcelo C. Santos, Paolo Zucchini, Barbara Contiero, Stefano Romagnoli, Celia R. Quirino, Isabel C. N. Cunha

**Affiliations:** 1 Department of Animal Medicine, Production and Health, University of Padua, 35020 Padua, PD, Italy; paolo.zucchini@unipd.it (P.Z.); barbara.contiero@unipd.it (B.C.); stefano.romagnoli@unipd.it (S.R.); 2 Norte Fluminense State University, Campos dos Goytacazes 28013-602, RJ, Brazil; mcs.vet@pq.uenf.br (M.C.S.); crq@uenf.br (C.R.Q.); cunhaicn@uenf.br (I.C.N.C.)

**Keywords:** canine, spermatozoa, polyunsaturated fatty acids, semen cryopreservation, EPA, DHA, fish oil

## Abstract

The objective of this study was to evaluate the effect of omega-3 oral supplementation on frozen–thawed semen quality in stud dogs. Nine healthy male dogs of various ages (2–11 years old), weights (10–36 kg), and breeds were included. Their daily diets were supplemented with salmon oil at 180 mg DHA/7 kg BW. Semen was collected before salmon oil oral administration (D0), after 90 days of daily administration (D90), and 90 days following suspension (D180). Samples underwent standard freezing procedures and were stored for at least 30 days; then, they were thawed at 37 °C for 1 min. Samples (n = 27) were assessed post-thawing for motility (MOT%), membrane integrity (HOST%), and viability (EN%). A second aliquot of the same samples underwent a thermoresistance test at 37 °C, with MOT% and EN% evaluated at 2 and 4 h post-thawing. Data were analyzed using repeated measures ANOVA, considering days of administration (D0, D90, D180), incubation times at 37 °C (T0h-T2h-T4h) and their interaction. Significance was set at *p* < 0.05. An increase in MOT% was observed after 90 days of fish oil administration (D0 = 56.67 ± 5.1% vs. D90 = 71.67 ± 5.1%, *p* < 0.05); however, this effect was not maintained at D180 (45.56 ± 5.1%). Similar results were observed for HOST and EN. During the thermoresistance test, a significantly higher MOT% was observed at T4h on D90 compared to D0 (*p* < 0.05). These findings indicate that daily salmon oil supplementation at 180 mg DHA/7 kg for 90 days improves semen motility, membrane integrity and viability, supporting a role for omega-3 fatty acids in semen undergoing cryopreservation.

## Simple Summary

Salmon oil is one of the richest sources of omega-3 polyunsaturated fatty acids. Its use as an oral supplement alongside various diets has been shown to improve many sperm characteristics, including motility and the ability to maintain functional sperm membranes in different animal species, such as bulls, dogs, sheep, poultry and horses. With this study, we evaluated the effect of a 3-month daily fish oil oral administration on the quality of sperm samples subjected to freezing and thawing, as well as the persistence of these effects 3 months after suspension of the supplementation. The results showed that dietary supplementation with salmon oil at a dosage of 180 mg DHA/7 kg body weight per day improved the motility, membrane integrity and viability of frozen–thawed sperm samples in the nine dogs included in this study. After 90 days of fish oil administration, an increase in the sperm motility was observed both after thawing and during a 4-h incubation at 37 °C. This improvement was also maintained in samples collected three months after the supplementation of salmon oil was suspended.

## 1. Introduction

Cryopreservation of male gametes allows for their long-term banking for an indefinite period of time, because of the very low temperature of liquid nitrogen (−196 °C). Metabolic activities of gamete cells are effectively arrested at those ultra-low temperatures, thus enabling their use after storage for a long time or following transportation over long distances [1]. Since the discovery of the cryoprotective properties of glycerol, a wide range of cryopreservation techniques and protocols have been developed [2]. Cryoprotectants in combination with several variables influencing the cryopreservation protocol, such as extender composition, freezing and thawing rates, semen concentration at freezing and semen dilution procedures, represent critical factors in the optimization of a freezing protocol. Collectively, the control of these key factors aims to reduce the dimensions of crystal ice into the cells. Ice crystal formation during the freezing process causes damage on the sperm cell membrane, thus reducing the post-thaw lifespan of spermatozoa. Membrane fluidity may play a role in sperm survival, since membrane fluidity imparts resistance to ice crystal-induced damage.

The composition of the sperm membrane provides resistance to thermal stress when characterized by a low polyunsaturated/saturated phospholipid fatty acids ratio [3,4,5,6]. Plasma membrane lipid composition varies considerably between normozoospermic and asthenozoospermic samples [7]. Numerous studies investigated the modulation of sperm membrane composition through dietary supplementation of polyunsaturated fatty acids (PUFAs). During cooling, the sperm plasma membrane needs flexibility; therefore, enrichment of the dietary supplementation with omega-3 PUFA, such as eicosapentaenoic acid (EPA) and docosahexaenoic acid (DHA), enhances membrane fluidity and resistance to cryodamage. PUFAs are synthesized in the body using alpha-linolenic acid (ALA) as a precursor [8,9]. Therefore, dietary supplementation with ALA or long-chain omega-3 PUFAs, most notably EPA and DHA, has been shown to provide significant health benefits [10].

Eicosapentaenoic acid (20:5 n-3, EPA), which is abundant in fish and algae, is a common dietary n-3 PUFA. EPA may attenuate oxidative stress, inflammation, cancers, hyperlipidemia, neurodegenerative diseases and other diseases, thereby exhibiting multiple beneficial effects on human health [8,11]. In dogs, n-3 PUFA supplementation has been associated with reductions in cardiovascular diseases, chronic renal failure, neoplastic processes, atopic dermatitis, and degenerative joint diseases, owing to their role as precursors of potent anti-inflammatory mediators, their immunomodulatory effect [11], and their capacity for counterbalancing alterations in nutrient metabolism that may lead to diabetes mellitus [10]. One of the richest sources of DHA and EPA is marine fish oil from deep-sea fish, such as salmon, tuna, menhaden, anchovies, herring and mackerel, which is commonly used [11]. There is some evidence that DHA and EPA added to the diet may improve sperm quality [12,13]. Oral administration of fish oil with various diets has been shown to improve sperm motility, viability and membrane integrity in bulls [14], dogs [15], sheep [16], poultry [17], and horses [18]. However, conclusive data are lacking regarding the effect of fish oil supplementation on sperm quality and motility following cryopreservation.

The objective of this study was to evaluate the effect of oral supplementation with salmon oil on the quality of canine semen by assessing frozen–thawed semen before, during 90 days of supplementation, and following 90-day withdrawal period. The effect of salmon oil supplementation was further investigated through sperm analysis performed immediately after thawing and after 2 h and 4 h of incubation at 37 °C.

## 2. Materials and Methods

### 2.1. Animals and Experimental Design

Nine healthy male dogs, belonging to various breeds (3 Flat-Coated Retrievers, 2 Shetland Sheepdogs, 1 Cocker Spaniel, 1 French Bulldog, 1 Boxer and 1 Labrador), weighing between 10 and 36 Kg and with an age interval within 2–11 years were included in this study. All dogs were privately owned, underwent general and andrological clinical examination prior to enrolment, which lasted between 1 and 2 months, and were of proven fertility based on previous normal litters. This study was approved by the ethics committee of Norte Fluminense State University (CEUA-UENF), protocol number 393/18. Data were collected between September 2017 and July 2018. Following the first clinical examination and semen collection (D = 0), all dogs received salmon oil supplementation, administered per os at a dose of 54 mg/Kg BW per day (Grizzly Salmon Oil, Grizzly Pet Products, LLC, Woodinville, WA, USA), where each 3.5 mL of the product (i.e., 0.7 tsp) contained the following: omega-3, 935 mg; omega-6, 95 mg; DHA, 355 mg; and EPA, 320 mg. This ensured the daily dose of 180 mg DHA/7 kg. Grizzly Salmon Oil™ is a liquid formula derived from wild Alaskan Salmon, which contains 40% more omega-3 when compared with other farmed salmon, as declared by the manufacturer and supported by the literature [19]. Supplementation was discontinued after 90 days, at which time a follow-up was scheduled (D = 90), with a third one 90 days later (D = 180). At each time point, dogs underwent a general physical examination, a semen collection and analysis as well as semen freezing.

### 2.2. Collection of Semen and Sperm Pre-Freezing Evaluation

Semen was collected via manual stimulation in the presence of a bitch in heat. The collection was fractioned and only the second sperm fraction was used. The volume of the second sperm fraction was measured on a graduated tube (mL). Before undergoing freezing, motility (MOT) and concentration were evaluated on a 20 µL aliquot of semen. Motility was expressed as percentage and subjectively estimated immediately after collection by placing 10 µL of sample between a slide and a coverslip pre-warmed at 37 °C, observed under light microscopy (Olympus, CX41 RF, Milan, Italy), at 400× magnification. Only spermatozoa moving in a straightforward direction for 1–2 sec were counted as motile, and the percentage of the total number of spermatozoa was estimated across 10 microscopic fields by two experienced operators.

Sperm concentration (sptz × 106/mL) was determined by means of a Bürker chamber (number of sptz counted × 100 × 1000 × the dilution factor used), followed by calculation of the total sperm count (sptz × 106 mL × semen volume = total sptz) [20]. Sperm morphology was assessed by using eosin–nigrosin staining (Eosin-Nigrosin, Minitube™, Tiefenbach, Germany) and classified as normal or with major or minor defects [21]. At least 200 spermatozoa were evaluated via bright-field microscope at 1000×. Only fresh semen samples with MOT ≥ 60%, total sperm count ≥ 200 × 106 and morphologically normal spermatozoa ≥ 60% [22] were included.

### 2.3. Semen Freezing

The semen freezing procedure was performed as previously described [23,24]. Immediately after collection, semen was centrifuged at 700× *g* for 6 min, the supernatant removed, and the pellet diluted at room temperature with Extender I (Tris-glucose-citric acid with 3% glycerol) to a concentration of 200 × 106 sptz/mL. After one-hour equilibration at +4 °C, samples were further diluted 1:1 with Extender II (1% Equex STM^®^ paste (Nova Chemical Sales, Scituate, MA, USA) and 7% glycerol) at +4 °C, resulting in a final concentration of 5% glycerol, 0.5% Equex STM paste and 100 × 106 spermatozoa/mL. Extended semen was immediately loaded into 0.5 mL straws (IMV Technologies, L’Aigle, France), sealed with Seal-Ease^®^ (Becton Dickinson and Company, Franklin Lanes, NJ, USA), placed horizontally 4 cm above the surface of liquid nitrogen for 10 min and then plunged into the liquid nitrogen.

### 2.4. Thawing and Post-Thaw Semen Evaluation

Straws were stored in a liquid nitrogen tank for at least one month, then thawed at 37 °C for 1 min in a waterbath; their content was transferred to a test tube and diluted 1:2 with a Tris buffer at 37 °C [23].

Semen was immediately divided into two aliquots. The first aliquot was used to assess motility (MOT), membrane integrity, and live/dead spermatozoa ratio. MOT was evaluated under light microscopy (400×), as previously described. Viability was determined using supravital eosin/nigrosin staining [20] and live/dead ratio of spermatozoa expressed as percentage (%). Sperm membrane integrity was assessed using the hypo-osmotic swelling test (HOST) [25], using 100 µL of a of 150 mOsm hypo-osmotic solution (0.735 g of sodium citrate, 1.351 g of fructose and 100 mL of distilled water) incubated at 37 °C for 20 min.

The second aliquot was used for the thermal resistance test (TRT) at 37 °C for 4 h, with MOT and EN evaluated at thawing (time 0), 2 and 4 h after thawing.

### 2.5. Statistical Analysis

All the analyses were conducted with SAS 9.4 (SAS Institute Inc. 2023, Cary, NC, USA). Normality of data was assessed using Shapiro–Wilks test. Normally distributed data were analyzed using an ANOVA model for repeated measures, considering days of fish oil administration and suspension (D0-90-180), hours at 37 °C incubation after thawing (T0h-T2h-T4h), and their interaction as fixed effect and dog as a repeated and random effect. Parameters considered were MOT and EN. As the HOST was obtained only at thawing (T0h), a one-way ANOVA model was used, considering time of fish oil administration (D0-90-180) as fixed effect. Non-normally distributed data were analyzed via non-parametric pairwise Wilcoxon test. Normally distributed data were presented as least squares mean (ls-mean) and SE, while non-normally distributed data were presented as median and interquartile range. Significance was set as *p* < 0.05.

## 3. Results

### 3.1. Motility (MOT), Viability (EN), and Hypo-Osmotic Swelling (HOST) Tests at Thawing

**Figure 1:**
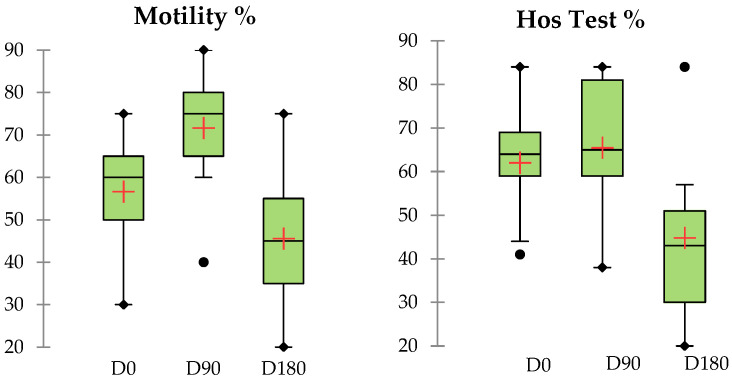
Box plots of motility % and HOST test % obtained from frozen–thawed semen samples of the patients enrolled in this study (n = 9) for the three observation times (D0, D90, D180).

**Figure 2:**
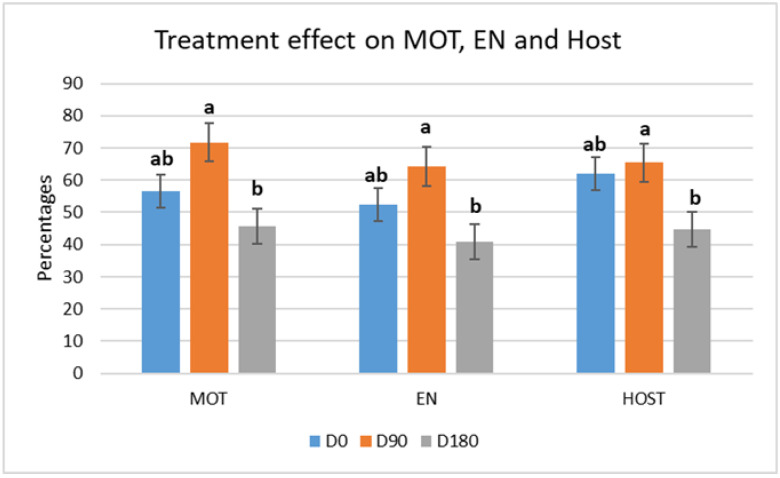
Total motility (MOT), viability (EN) and membrane integrity (HOST) of frozen–thawed spermatozoa at thawing for the three observation times before (D0, blue bars), during (D90, orange bars), and after suspension (D180, grey bars) of fish oil administration. Different letters (a, b, ab) mean a statistically significant difference (*p* < 0.05). Data are reported as ls-means and SE.

Figure 1 and Figure 2 report the descriptive statistics and analysis of data of MOT, EN, and HOST tests at thawing on the three observation times, before, during and after suspension of fish oil administration (D0-90-180).

An increase of MOT from D0 to D90 was observed (56.67 ± 5.1% vs. 71.67 ± 5.1%, respectively, *p* < 0.05); however, at D180, MOT returned to values lower than those observed before fish oil administration at T0 (MOT = 45.56 ± 5.1%). The same pattern was observed for EN and HOST.

### 3.2. Motility (MOT) and Viability (EN) During the Thermoresistance (TRT) Test

The results of the TRT test for MOT and EN performed at the three time points relative to fish oil administration (D0-D90-D180) are reported in Table 1 and Table 2, respectively. At thawing, MOT was higher at D0 and D90 with respect to D180 (*p* < 0.05). At 2 h of incubation, this difference could not be observed. However, at T4h of incubation, the situation was reversed, motility being higher at D90 than D0 (*p* < 0.05), with D180 mean values being in between the ones at D0 and D90 (*p* < 0.05).

The same pattern could be observed for EN during the incubation time (see Table 2). No differences were found for D180, with MOT and EN percentages being fairly stable during all the 4-h incubation times.

### 3.3. Sperm Morphology During D0-D90-D180

Sperm morphology results are presented in Table 3. The percentage of normal spermatozoa increased between D0 (57.5%) and D90 (80%) (*p* < 0.05) and remained high at D180 (84%). A similar (but inverse) pattern was observed for the percentage of spermatozoa with minor defects, which differed between D0 and D90 but not between D90 and D180 (Table 3). No differences were observed for major defects.

## 4. Discussion

In this study, the effects of oral supplementation with salmon oil on semen cryopreservation in stud dogs were investigated by evaluating classical post-thaw parameters, which includes motility, viability and membrane integrity. The first notable result was a mean increase of 15% in sperm motility at thawing following three months of daily salmon oil administration (56.67 ± 5.1% vs. 71.67 ± 5.1%, respectively, *p* < 0.05). However, this improvement was not sustained three months after fish oil supplementation ended, as sperm motility at thawing dropped below those observed before fish oil administration (45.56 ± 5.1%). A similar pattern was observed for viability and membrane integrity, as assessed via the eosin/nigrosin and hypo-osmotic swelling tests, with increased percentages of viable, intact sperm after three-month supplementation and a subsequent decrease after salmon oil suspension. The second relevant result emerged from the thermoresistance test. After three months of fish oil administration, motility was higher not only at thawing but also after 2 and 4 h of semen incubation at 37 °C. Following three months of fish oil suspension, some residual effects were still observed during thermoresistance test, with a lower mean motility percentage detected at thawing. However, values remained stable and within acceptable motility percentages throughout the four-hour incubation period, compared to the more pronounced decline observed at earlier observation times.

Freezing–thawing of spermatozoa is currently a widely used procedure that has brought beneficial effects in canine-assisted reproductive technologies. Interest in this technique has grown, especially due to the development of practical and effective freezing protocols [23] that allow for long-term storage and facilitate breeding selection through genetic preservation and global transport and shipment of the frozen material. Frozen–thawed semen typically exhibits a marked reduction in sperm motility, resulting from osmotic, thermal, and mechanical stress affecting the sperm membrane throughout the process. Conception rates depend on the post-thaw quality of canine semen and are generally lower than those achieved with fresh semen [26], although transcervical insemination techniques have simplified intrauterine deposition of frozen–thawed semen and improved pregnancy rates [27,28].

Reactive oxygen species (ROS) are highly reactive molecules that contains oxygen radicals. Their production results from the oxidative metabolism of the cell, and spermatozoa are particularly sensitive to ROS because they have a limited antioxidant capacity. Sperm cells are especially vulnerable to the detrimental action of ROS during the freezing process, when excessive ROS are generated in response to the oxidative stress, and this overproduction is not adequately counterbalanced by the enzymatic antioxidant activity of the cell [29,30]. The sperm cell is a highly specialized cell whose membrane plays essential roles in reproductive processes, such as motility, capacitation, acrosome reaction and sperm-zona pellucida binding. Recent studies highlighted the role of the lipid composition of the mammalian sperm membrane, especially the effects of its composition in governing and modulating physiological processes during spermatogenesis [31]. The sperm membrane is composed of a combination of three classes of lipids: phospholipids, sterols and glycolipids [32]. Among these, phospholipids received particular attention because they are the main membrane lipids enriched in PUFA. PUFAs are essential for membrane fluidity; however, they are the ones most subjected of ROS and lipid peroxidation. Docosahexaenoic acid (DHA) 22:6 (ω-3) and docosapentaenoic acid (DPA) 22:5 (ω-6) are two major phospholipid components located at the sn-2 position, and their proportions in the sperm membrane vary among species.

Lipid markers of sperm motility identified through Matrix-Assisted Laser Desorption/Ionization Mass Spectrometry (MALDI-MS) include phosphatidylcholine 38:4, phosphatidylcholine 36:1, phosphatidylethanolamine 34:4, glycerophosphatidic acid 36:4, plasmanyl and plasmenyl [7]. Distinct differences in membrane lipid composition have been observed between motile and astenospermic samples, indicating a correlation between sperm motility and the lipid profile [33]. In particular, the lipid composition of the sperm membrane and the concentration of polyunsaturated fatty acid DHA are positively associated with sperm motility and post-thaw viability, whereas monounsaturated fatty acids (MUFAs) are negatively correlated, thus suggesting that the fatty acid content can be used as a predictor of cryopreservation tolerance [33]. Furthermore, the high cholesterol-to-phospholipid ratio and the high PUFA levels appear to contribute to the greater resistance of canine sperm to freezing procedures. A high cholesterol level combined with a low PUFA content may lead to an excessive membrane rigidity, which may have deleterious consequences during freezing procedures, as lipid peroxidation may increase ROS production and cause membrane damage [34]. The beneficial effects of ω-3 and ω-6 dietary supplementation on reproductive male parameters have been well documented [15,16,17,18,35,36,37,38]. The incorporation of fatty acids into the diet can alter the lipid composition of the sperm membrane, which, in turn, may improve sperm cell motility, viability and resistance to stressors such as temperature fluctuations and osmotic changes.

Our results on motility and viability are similar to results obtained by Khoshvaght et al. [14], who found an increase in the percentage of live sperm, an effect that was further enhanced when vitamin E was added to the fish oil [38]. In stallions, supplementation with vitamin E, L-carnitine and selenium in combination with omega-3 and -6 resulted in increased sperm motility and preserved integrity of the acrosomes and plasma membrane in fresh and frozen/thawed semen [39]. These authors concluded that the increase in sperm motility was primarily due to the use of antioxidants, whereas improved membrane integrity was mainly attributed to the higher concentration of polyunsaturated fatty acids associated with the antioxidants rather than the omega-3 and -6 compounds alone [39]. Consequently, in stallions, isolated supplementation with omega-3 and -6 had limited effects on semen quality [39,40].

The influence of PUFAs on the freezability and post-thaw motility of canine spermatozoa remains debated [33,41]. Failure to demonstrate a positive effect of fish oil on canine semen quality may depend on factors such as the type and composition of fish oil, duration of dietary supplementation, and the specific freezing–thawing protocol employed. We hypothesized that the dietary intake of salmon oil increases post-thaw sperm motility after 90 days of supplementation, consistent with our previous study, which showed an increase in sperm concentration and motility in fresh samples, as well as an increase in testosterone levels in dogs fed salmon oil for 90 days [15]. Doppler velocimetric parameters, including peak systolic velocity and end diastolic velocity of the supratesticular and marginal arteries, were also improved [42]. We hypothesized that high PUFA levels in the sperm membrane, particularly DHA, provide protection against the effects of lipid peroxidation, offsetting the action of free radicals [43], thereby improving sperm motility. Increased sperm motility in frozen–thawed ram semen following fish oil supplementation has been reported [16]. Moreover, in monkeys, the DHA concentration was significantly higher in the sperm tail than in the head (19.6% vs. 1.1%, respectively), suggesting a more pronounced involvement in sperm motility than in acrosomal function [44]. Many authors note the dual role of EPA as both a pro-oxidant, due to its high degree of unsaturation, and an antioxidant, with recent evidence of its antioxidant activity via modulation of mitochondrial function [45]. Further studies are needed in order to clarify EPA’s role, particularly in the mitochondrial membrane and its overall antioxidant function in sperm cell.

In a previous study, we demonstrated the fish oil residual effect on motility, morphology and membrane integrity parameters, not only during three months of fish oil supplementation but also three months after the cessation of fish oil administration, as assessed through semen analysis in fresh semen samples [15]. In the present study, some effects are still observable in the thermoresistance test, where the motility of frozen–thawed semen after 4 h of incubation remained high both during fish oil administration and 3 months after its discontinuation. The thermoresistance test was performed to assess the ability of sperm to survive and maintain motility and viability, not only at thawing but also for several hours after incubation at 37 °C [22]. These conditions mimic the temperature and duration in the female vaginal tract during colonization of the uterine crypts. Spermatozoa do not acquire fertilizing capacity immediately after insemination, as capacitation is required first. However, frozen–thawed semen may undergo subtle membrane damage, potentially inducing capacitation-like changes that reduce the sperm lifespan within the vaginal tract. The increased membrane integrity observed during fish oil supplementation in our study could account for the enhanced thermoresistance observed both after 90 and 180 days of fish oil administration. Very few studies have investigated the duration of the effects of fish oil on the lipid composition of cell membranes, and almost none have focused on the timing of lipid incorporation into sperm membranes. Hansen et al. (1998) [46] examined the interval between the rise and decline in serum omega-3 fatty acid concentrations during and after the cessation of dietary supplementation, and they postulated that DHA levels remained elevated for seven weeks after the cessation of supplementation. Considering that spermatogenesis and spermiogenesis require over 60 days for sperm release from the seminiferous tubules, this finding may explain the residual effects observed in semen collected and frozen three months after the suspension of fish oil administration. In another three-month study on the effects of daily administration of a DHA-rich supplement, a significant improvement in the fatty acid profile of canine erythrocytes was observed after one week; after discontinuation, the ω-3 fatty acid levels declined gradually over four weeks, without returning to baseline [47]. As erythrocyte production is a considerably faster process than spermatogenesis, these findings support the hypothesis that a change in the fatty acid profile of sperm cell membranes may persist for a prolonged period after the discontinuation of the supplementation. Further studies are, however, required to clarify the duration and dynamics of PUFA incorporation into sperm membranes following a dietary supplementation.

In dogs, male infertility is characterized by an increase in defective spermatozoa beyond the threshold of 20% [48]. The percentage of morphologically normal spermatozoa increased at D90 and remained higher than baseline until D180, demonstrating that salmon oil supplementation improved sperm morphological parameters. The germinative cells and Sertoli cells have an active lipid metabolism that reshapes the fatty acid complement, leading to elongation and desaturation processes that are essential during spermatogenesis and possibly during the maturation of spermatozoa [15]. This metabolic activity may underlie the improvement in semen parameters, including the higher proportion of normal spermatozoa. A reduction in sperm defects was also reported by Attaman et al. (2012) [49] in men receiving dietary omega-3 supplementation. In our study, sperm morphology after freezing was not affected by the supplementation. Morphological alterations during cold storage are known to occur due to changes in membrane structure [50]. Among the polyunsaturated fatty acids, DHA accounts for approximately 48–52% of the total fatty acid content in spermatozoa with normal morphology [51].

Despite the encouraging evidence of improved sperm parameters observed in this study, a deeper understanding of the mechanisms of action of orally administered EPA and DHA presumably requires more specific analytical approaches. Fluorescent probes and flow cytometric analysis capable of identifying acrosomal membrane integrity and lipid peroxidation status should be employed. Furthermore, characterization of the sperm membrane lipidic profile before and after fish oil supplementation would provide clearer knowledge into the specific site of action of the sperm cell compartments, more clearly revealing the cause of the observed improvement. This is particularly relevant since most of the available studies have been conducted on patients with normal or unknown fertility. Focusing on subjects with diagnosed hypofertility due to hypozoospermia, teratozoospermia or reduced motility could yield more conclusive evidence on the potential benefits of fish oil supplementation for canine semen quality.

Lastly, a lack of a balanced and standardized diet across the enrolled subjects may have limited the ability to assess the effects of omega-3 supplementation. The ratio of n-3 to n-6 fatty acids and their total dietary intake is crucial for maintaining wellbeing and health. The National Research Council recommends an n-6–n-3 ratio of 2.6:1 to 26:1 [11,52]. Although omega-3 fatty acid supplementation has demonstrated clinical utility in several veterinary areas of specialization, including dermatology, orthopedics, oncology, cardiology, endocrinology, nephrology, cognition and behavior, there is currently no clear consensus in the literature regarding its application in improving reproductive outcomes in female or male dogs undergoing breeding programs.

## 5. Conclusions

Dietary supplementation with salmon oil at a dosage of 180 mg DHA/7 kg BW per day for 90 days improved the motility, membrane integrity and viability of frozen–thawed semen in the nine dogs in our study. After 90 days of fish oil oral administration, we detected an increase in the motility both at thawing and during the 4-h thermoresistance test, and these effects persisted in samples collected three months after the discontinuation of salmon oil supplementation. Sperm morphology was, likewise, improved during and after fish oil administration, with an higher percentage of normal spermatozoa detected after 90 days of treatment and maintained for an additional 90 days, paralleling the results of the thermoresistance test. Further studies are needed to confirm these findings using more advanced analytical techniques, such as Computer-Assisted Sperm Analysis for motility and flow cytometry for viability, as well as to investigate whether salmon oil supplementation at the proposed DHA dosage may improve acrosomal membrane quality and cryotolerance by reducing the effects of lipid peroxidation. Moreover, further research is needed to evaluate the effects of omega-3 supplementation in dogs maintained on standardized diets to reduce the variability in nutritional backgrounds.

## Figures and Tables

**Table 1:** Thermoresistance test (TRT) during incubation of thawed semen at 37 °C for 2 and 4 h post-thawing. Results during incubation at T0h, T2h, T4h, before (D0), during (D90), and after suspension (D180) of salmon oil administration are reported as least square-mean ± SE. Different Latin letters among columns (a, b, ab) and different Greek letters among rows (α, β) mean significantly different values (*p* < 0.05) for time of fish oil administration and suspension (D0-90-180) and hours after thawing (T0h-T2h-T4h), respectively.

MOT (%)			
	T0h	T2h	T4h
D0	56.7 ± 5.1 abα	32.1 ± 6.3 β	16.7 ± 5.9 bβ
D90	71.7 ± 5.1 aα	46.7 ± 6 β	37.8 ± 5.9 aβ
D180	45.6 ± 5.1 b	38.9 ± 6	35.9 ± 5.9 ab

**Table 2:** Ls-mean and SE of live/dead ratio (EN) of frozen–thawed semen of 9 dogs fed an oral supplementation of salmon oil for 90 days. Semen aliquots underwent a thermal resistance (TRT) test after thawing (T0h), and at 2 h and 4 h of incubation at 37 °C before (D0), during (D90) and three months following suspension of oral supplementation (D180). Different Latin letters among columns (a, b, ab) and different Greek letters among rows (α, β) indicate significantly different values (*p* < 0.05) for salmon oil administration and suspension (D0-90-180) and hours of incubation of thawed semen (T0h-T2h-T4h), respectively.

EN (%)			
	T0h	T2h	T4h
D0	52.3 ± 5.95 abα	34.8 ± 5.3 β	30.8 ± 6.4 β
D90	64.2 ± 5.95 aα	38.3 ± 5.1 β	32.7 ± 6.1 β
D180	40.8 ± 5.95 b	36 ± 5.2	38.1 ± 6.1

**Table 3:** Median and interquartile range of the sperm morphology of 9 dogs supplemented orally with salmon oil for 90 days (D0-D90) and 3 months following supplementation suspension (D180). Different letters in the same row (a, b) indicate a statistically significant difference. *p*-values are reported.

	D0	D90	D180	*p*-Value
Normal Spermatozoa (%)	57.5 (45–65) b	80 (75–86) a	84 (83–87) a	0.005
Major Defects (%)	6.5 (4.5–20)	4 (2–7)	6 (3–8)	0.32
Minor Defects (%)	36 (26–40.5) a	13 (11–19) b	10 (9–11) b	0.003

## Data Availability

Data are available upon requests.
